# Prognostic impact of pathologically confirmed venous infiltration during upfront pancreatectomy: multicenter survival analysis

**DOI:** 10.1007/s00423-026-04041-2

**Published:** 2026-04-09

**Authors:** Giampaolo Perri, Riccardo Guastella, Muyue Liu, Elena Antelmi, Nicola Canitano, Jianzhen Lin, Riccardo Pellegrini, Samuele Grandi, Zipeng Lu, Laura Mastrangelo, Domenico Bassi, Elio Jovine, Kuirong Jiang, Umberto Cillo, Giovanni Marchegiani

**Affiliations:** 1 Hepato-Pancreato-Biliary and Liver Transplant Surgery Unit, Department of Surgical, Oncological and Gastroenterological Sciences (DiSCOG), Università degli Studi di Padova, Padova, Italy; 2 Pancreas Center, The First Affiliated Hospital of Nanjing Medical University, Institute of Pancreas, Nanjing Medical University, Nanjing, China; 3 Department of Surgery, AOU Sant’Orsola Malpighi, IRCCS Azienda Ospedaliera Universitaria, Bologna, Italy; 4 Chirurgia Epato-Bilio-Pancreatica e dei Trapianti di Fegato, Dipartimento di Scienze Chirurgiche Oncologiche e Gastroenterologiche - DISCOG, Università di Padova, Via Giustiniani 2, Padova, 35128 Italy

**Keywords:** Pancreatic Neoplasms, Pancreatectomy, Portal Vein, Vascular Resection, Chemotherapy

## Abstract

**Purpose:**

Pancreatectomy with venous resection (PVR) for resectable pancreatic ductal adenocarcinoma (PDAC) may be preoperatively planned or intraoperatively required due to suspected vascular involvement. The prognostic impact of pathologically confirmed venous infiltration (PVI) in upfront PVR remains unclear. This study assesses such impact on overall survival (OS).

**Methods:**

Patients who underwent upfront PVR for PDAC were identified from a prospectively maintained database across three high-volume institutions. Clinical and pathological variables, including PVI status, were collected. Survival outcomes and predictors were analyzed using Cox regression models.

**Results:**

A total of 295 patients underwent upfront PVR. Segmental end-to-end reconstruction (Type III) was the most common approach (69%). PVI was confirmed in 66% of cases. Adjuvant therapy was administered to 69% of patients. Median OS was 32 months for PVI-negative vs. 21.1 months for PVI-positive patients (HR 1.49, 95% CI: 1.02–2.17, *p* = 0.037). One- and three-year survival rates were significantly lower in the PVI-positive group. Among PVI-positive patients, OS was 7 months without adjuvant therapy and 29.5 months with, aligning with outcomes in PVI-negative patients who received adjuvant treatment (HR 1.51, *p* = 0.104). Independent predictors of OS included PVI (HR 1.67), age ≥ 75 (HR 1.93), N2 status (HR 1.89), total pancreatectomy (HR 2.42) and adjuvant therapy (HR 0.33).

**Conclusions:**

More than one-third of patients undergoing upfront PVR for suspected venous infiltration are overtreated as they lack pathologically confirmed vessel involvement. Conversely, when PVI is present, survival is significantly impaired. The successful delivery of adjuvant chemotherapy in case of PVI ameliorates outcomes as in cases lacking venous involvement.

**Supplementary Information:**

The online version contains supplementary material available at 10.1007/s00423-026-04041-2.

## Introduction

Pancreatic ductal adenocarcinoma (PDAC) is among the most lethal malignancies, with an overall 5-year survival rate not superior to 10%, mainly due to late diagnosis and limited effective treatment options [1–4]. The combination of radical surgical resection and chemotherapy remains the best therapeutic chance, yet only 15–20% of patients are eligible for it due to local vascular involvement or distant metastases [2, 5, 6]. The involvement of the portal vein (PV) and superior mesenteric vein (SMV) is particularly common in PDAC of the pancreatic head and body, due to close anatomical proximity. While historically considered a contraindication to proceed with surgery, venous invasion is now addressed in selected patients with localized disease by performing pancreatectomy with venous resection (PVR), aiming to achieve a margin-negative (R0) resection [7–10]. Several large series and meta-analyses have indeed confirmed that PVR, though associated with increased operative complexity, is technically feasible and oncologically justified at expert centers without increasing postoperative complications [11, 12]. Neoadjuvant chemotherapy followed by possible PVR has become the standard of care for borderline resectable PDAC, as outcomes are ameliorated by patient selection and tumor response to treatment [13, 14]. However, the same concept does not apply yet to resectable PDAC. In this setting, the role of neoadjuvant chemotherapy still is controversial. According to current guidelines [15, 16] it is usually reserved to clinical trials, resulting in the majority of resectable PDAC undergoing upfront surgery. Contraindications to neoadjuvant therapy may include poor performance status, uncontrolled obstructive jaundice, severe comorbidities, or patient refusal of systemic treatment. Of note, according to NCCN [15], the anatomical definition of resectable PDAC includes tumors with < 180° infiltration of PV/SMV, which should therefore undergo upfront PVR as well. Finally, several patients with borderline resectable PDAC and systemic contraindication to neoadjuvant therapy may also be treated with upfront PVR.

In the context of upfront PVR, the prognostic role of a pathologically confirmed venous invasion (PVI) has been scarcely defined but might direct the decision making. Indeed, an intraoperatively detected macroscopic adherence of the tumor to PV or SMV does not always correspond to true microscopic invasion [17, 18]. However, once PVR is performed, the actual oncologic significance of PVI and its implication on survival remain controversial, especially in patients who did not receive prior chemotherapy. In a multicenter retrospective study of 406 patients who underwent PVR, a PVI was confirmed in only 56.7% of specimens, but once this was present the 5-year overall survival was only 20% compared to 33% in patients without PVI [19]. These results suggest that nearly half of patients receiving upfront PVR might undergo overtreatment. Conversely, confirmed PVI might be a negative prognostic factor in the other half, advocating for prior chemotherapy.

This study aims to clarify the oncological significance of PVI in patients undergoing upfront PVR for PDAC, focusing on its impact on overall survival (OS) and the role of adjuvant chemotherapy in modulating its effect.

## Materials and methods

### Study design and patient selection

This is a retrospective, multicenter cohort study conducted at three high-volume Institutions in Italy and China. Included patients underwent upfront PVR for histologically confirmed PDAC between January 2015 and December 2023. Each center prospectively maintained a surgical database from which eligible patients were identified. Given the retrospective and anonymized nature of the study, a specific informed consent for this analysis was waived. Patients were eligible if they had undergone PVR either preoperatively planned, based on radiological suspicion, or performed upon intraoperative macroscopic evidence of vascular involvement. Patients who had received neoadjuvant chemotherapy or radiotherapy were excluded in order to avoid potential confounding related to tumor downstaging and treatment-induced fibrosis, which may alter the pathological appearance of the venous wall and affect the assessment of true venous invasion at final pathology. Patients with non-PDAC diagnosis at final pathology or with incomplete pathological or survival data were also excluded from the analysis.

### Surgical procedures

Patients underwent pancreaticoduodenectomy (PD), distal pancreatectomy (DP), or total pancreatectomy (TP) based on tumor location and local extension. Venous resection was classified according to the International Study Group of Pancreatic Surgery (ISGPS) definitions [20]. Partial venous resection with direct venorrhaphy (type I), partial resection with patch reconstruction (type II), segmental resection with direct veno-venous anastomosis (type III), and segmental resection with interposition graft (type IV) were adopted based on the extent of venous involvement and intraoperative judgment. Management of the splenic vein, including ligation, preservation, or reimplantation, was determined according to tumor location and intraoperative findings. In case of reimplantation, the splenic vein was reimplanted into the portal vein or superior mesenteric vein using an end-to-side anastomosis after completion of the venous reconstruction, in order to reduce the risk of left-sided portal hypertension. Intraoperative anticoagulation was administered in the majority of patients to reduce the risk of thrombosis during venous reconstruction. In addition, the reconstructed vein was routinely flushed with heparinized saline before completion of the anastomosis. Similarly, the technique of vascular reconstruction and the type of pancreatic, biliary, and enteric anastomoses were decided at the discretion of the operating surgeon, depending on anatomical and technical considerations.

### Pathological examination

All surgical specimens were evaluated by experienced pathologists at the respective centres. Histopathological analysis included assessment of tumor size, lymph node involvement, resection margin status, and the presence of PVI, defined as the microscopic infiltration of the venous wall by tumor cells. Margin status was classified according to established criteria, with R1 defined as the presence of tumor cells within 1 mm of the resection margin, as proposed by Campbell et al. [21].

### Definition of clinical outcomes

The primary endpoint of the study was OS, defined as the time from the date of surgery to death from any cause or the date of last follow-up. Secondary outcomes included the rate of R0 resection, lymph node status according to the AJCC 8th edition classification [22], and the administration of adjuvant therapy.

Postoperative complications were classified according to the Clavien–Dindo grading system [23], and postoperative mortality was defined as any death occurring within 30 days after surgery or during the same hospital admission. Survival analyses also evaluated 1- and 3-years survival rates, stratified by the presence or absence of PVI. Due to the small number of patients with available follow-up beyond three years, 5-year survival rates were not included to avoid overinterpretation of unstable data. The impact of adjuvant therapy on survival outcomes among patients with PVI was specifically assessed. Additionally, multivariable analysis was performed to identify independent predictors of OS.

### Adjuvant therapy and follow-up

Adjuvant chemotherapy was recommended according to multidisciplinary team evaluations based on postoperative pathological findings and patient performance status. In general, it was administered to patients with adequate postoperative recovery and performance status, particularly in the presence of adverse pathological features such as lymph node positivity or margin involvement. Major contraindications to adjuvant therapy included inadequate postoperative recovery, poor performance status, or significant medical comorbidities. Specific chemotherapy regimens and schedules were determined individually by each treating center according to institutional protocols.

Postoperative follow-up included clinical assessments, tumor marker measurements, and cross-sectional imaging at regular intervals, in accordance with each institution’s standard practice.

### Statistical analysis

Statistical analyses were performed using Jamovi web platform (version 2.6.13) [24]. Continuous variables were presented as medians with interquartile ranges (IQR) and compared using the Mann–Whitney U test, while categorical variables were expressed as frequencies and percentages and compared using the chi-square or Fisher’s exact test as appropriate. Overall survival was estimated using the Kaplan–Meier method, and survival curves were compared by the log-rank test. Univariable and multivariable Cox proportional hazards regression models were used to identify independent predictors of overall survival. Variables achieving a p-value < 0.05 in univariable analysis were entered into the multivariable model. Hazard ratios (HRs) with corresponding 95% confidence intervals (CIs) were reported, and statistical significance was defined as a two-sided p-value of less than 0.05.

## Results

### Patients and perioperative characteristics

A total of 295 patients met the inclusion criteria and were included in the final analysis. Perioperative data of patients are shown in Table 1. The median age at the time of surgery was 66 years (IQR: 14), and 48% of patients were female (*n* = 142). Based on preoperative imaging, 81% of tumors were classified as radiologically resectable (*n* = 239), while 19% were borderline resectable (*n* = 56). The most frequently performed surgical procedure was PD, accounting for 70% of cases (*n* = 206). The ISGPS type III was the most common venous resection technique, performed in 69% of patients (*n* = 202). The median operative time was 330 min (IQR: 123). Intraoperative administration of systemic heparin was documented in 86% of cases (*n* = 255). As for the vascular reconstruction, the majority were performed between two segments of the SMV (*n* = 168; 57%), followed by PV to SMV (*n* = 87; 29%), and PV to PV (*n* = 40; 14%). At pathological examination, the median tumor diameter was 35 mm (IQR: 13). Based on the AJCC 8th edition staging system [22], 34% of tumors were classified as stage IIB (*n* = 100), while 44% were stage III (*n* = 131). N2 lymph node status was present in 24% of cases (*n* = 70). Resection margins were microscopically positive (R1) in 58% of patients (*n* = 172), while R0 resection was achieved in 42% (*n* = 123). PVI was confirmed in 196 patients (66%). Clinicopathological characteristics stratified by PVI status are reported in Supplementary Table. Among these patients, lymph node status was distributed as follows: N0 in 51 patients, N1 in 95 patients, and N2 in 50 patients. Adjuvant chemotherapy was administered in 69% of cases (*n* = 204). Among the 204 patients who received adjuvant therapy, the most commonly administered regimens were gemcitabine-based chemotherapy (132 patients, 65%), followed by fluoropyrimidine-based regimens (42 patients, 20%) and FOLFIRINOX-based regimens (20 patients, 10%). Other less frequently used regimens accounted for 10 patients (5%). Major postoperative complications, defined as Clavien–Dindo grade III or higher, occurred in 33% of patients (*n* = 98). The most frequent complications included clinically relevant postoperative pancreatic fistula (POPF grade B/C) in 51 patients (17%), post-pancreatectomy hemorrhage (PPH grade B/C) in 29 patients (10%), and delayed gastric emptying (DGE grade B/C) in 17 patients (6%). Major postoperative complications occurred in 27% of patients who subsequently received adjuvant chemotherapy compared with 46% of those who did not receive adjuvant therapy (*p* = 0.002). The 30-day and 90-day postoperative mortality rates were 2% and 6%, respectively.

**Table 1:** Perioperative characteristics of all patients (2015–2023) (*N* = 295)

Characteristics	Total, n (%) (*N* = 295)
Preoperative	
Age, Median (IQR), y	66 (14)
Female Sex, n (%)	142 (48)
ASA Score ≥ 3, n (%)	86 (29)
Preoperative biliary drainage, n (%)	36 (12)
Ca 19.9, Median (IQR), U/mL	179 (541)
Tumor Resectability (NCCN), n (%):	
Resectable	239 (81)
Borderline Resectable	56 (19)
Lesion Site, n (%):	
Head	237 (80)
Istmus	4 (1)
Body-Tail	43 (15)
Multifocal	11 (4)
Intraoperative	
Surgery Type, n (%):	
Pancreaticoduodenectomy	206 (70)
Distal Pancreatectomy	39 (13)
Total Pancreatectomy	50 (17)
Operative Time, Median (IQR), min	330 (123)
Venous Resection, n (%):	
Tangential	72 (24)
Segmental	223 (76)
ISGPS Type, n (%):	
I. (Tangential with Primary Closure)	48 (16)
II. (Tangential with Peritoneal Patch)	24 (8)
III. (T-T with Primary Anastomosis)	202 (69)
IV. (T-T with Interposition Graft)	21 (7)
Site of Anastomosis, n (%):	
PV-PV	40 (14)
SMV-SMV	168 (57)
PV-SMV	87 (29)
Intraoperative Heparin (Reconstruction Phase), n (%)	255 (86)
Pathology	
Tumor Diameter, Median (IQR), mm	35 (13)
AJCC Stage, n (%):	
IA	4 (1)
IB	26 (9)
IIA	32 (11)
IIB	100 (34)
III	131 (44)
N/A	2 (1)
Number of Harvested Lymphnodes, Median (IQR)	20 (14)
Lymphnode Ratio, Median (IQR), %	8 (18)
N Status, n (%):	
N0	84 (28)
N1	141 (48)
N2	70 (24)
R Status, n (%):	
R0	123 (42)
R1	172 (58)
Vein Invasion Confirmed by Pathology, n (%)	196 (66)
Postoperative	
Major Morbidity (Clavien-Dindo ≥ 3), n (%)	98 (33)
Mortality (30-days/in-hospital), n (%)	6 (2)
Mortality (90-days), n (%)	17 (6)
Adjuvant Therapy, n (%)	204 (69)
Gemcitabine-based regimens	132 (65)
Fluoropyrimidine-based regimens	42 (20)
FOLFIRINOX-based regimens	20 (10)
Other regimens	10 (5)

*ASA* indicates American Society of Anesthesiologists, *ISGPS* International Study Group for Pancreatic Surgery, *PV* Portal Vein, *SMV* Superior Mesenteric Vein, *AJCC* American Joint Committee on Cancer

### Overall survival and impact of venous infiltration

**Fig. 1:**
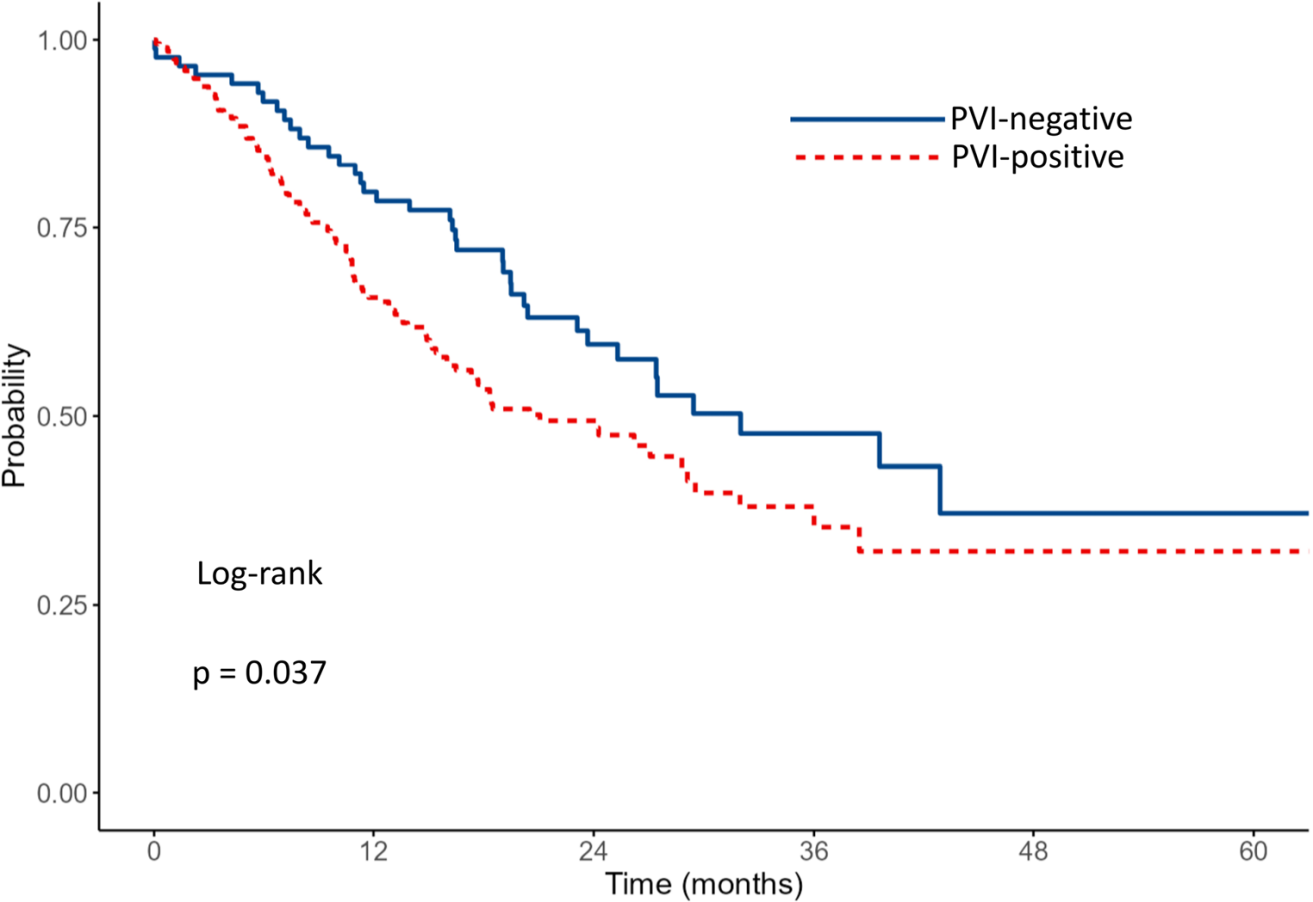
Kaplan–Meier survival curves comparing OS between patients with and without PVI

**Fig. 2:**
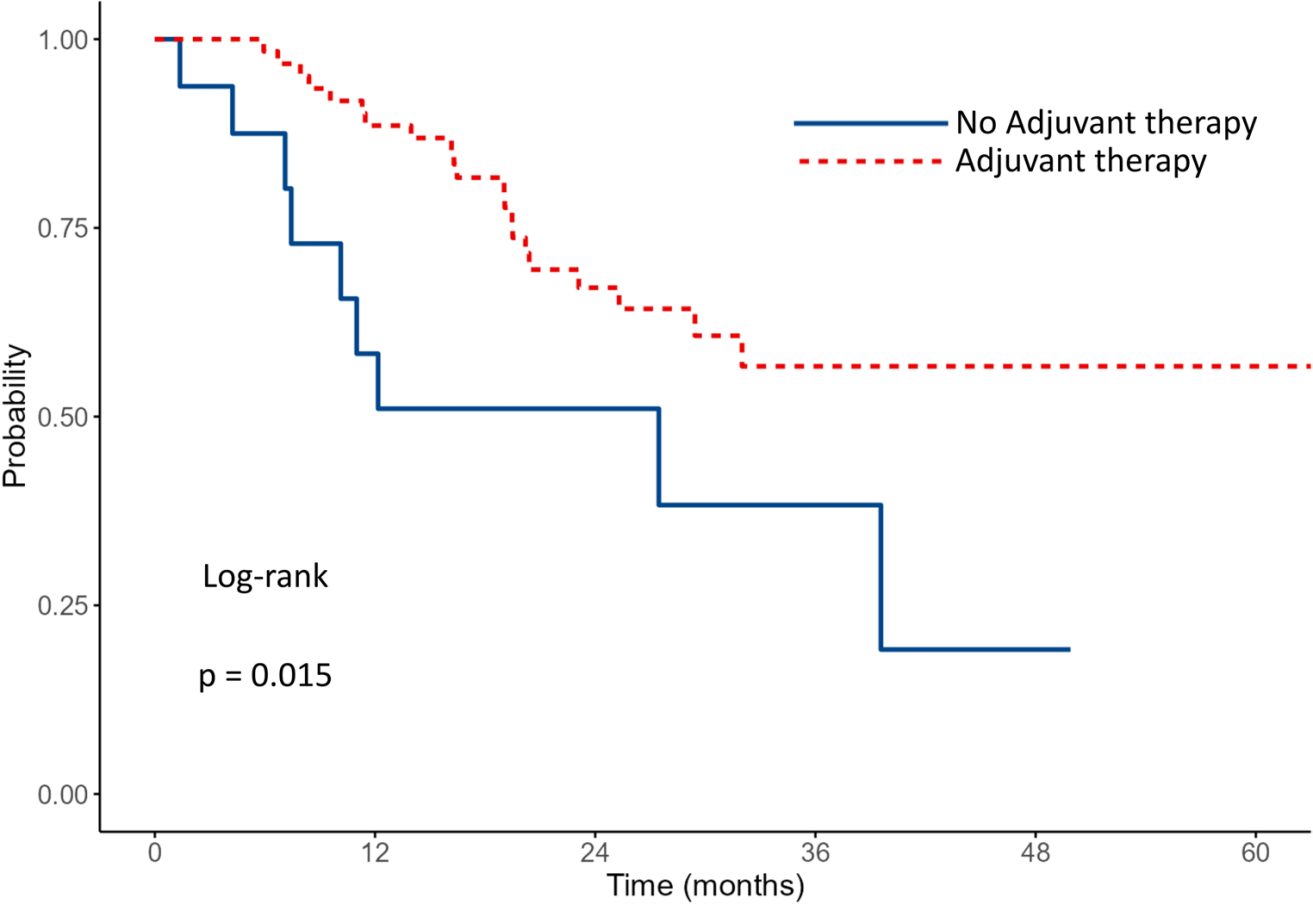
Kaplan–Meier survival curves comparing OS between PVI-negative patients with and without adjuvant therapy

**Fig. 3:**
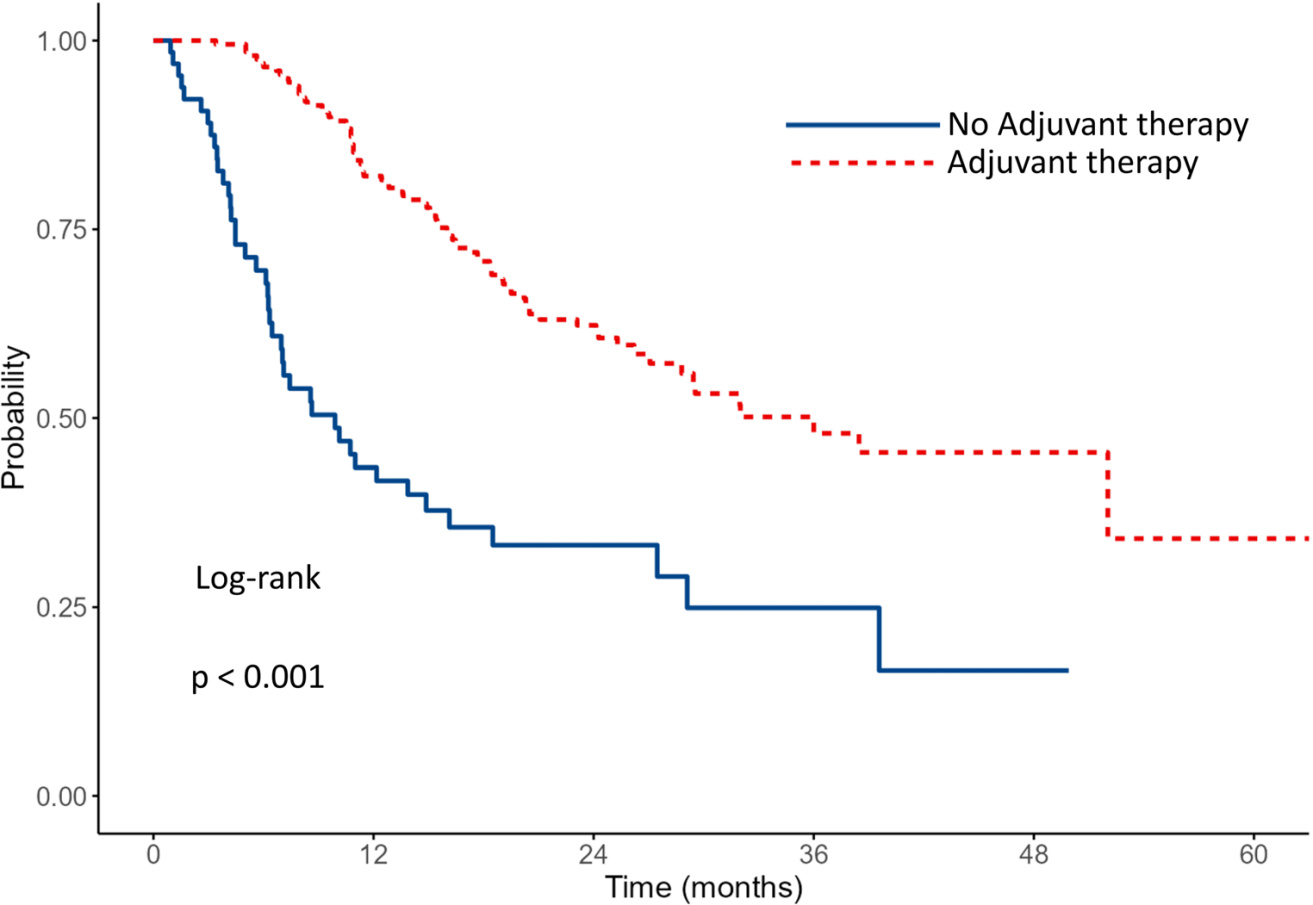
Kaplan–Meier survival curves comparing OS between PVI-positive patients with and without adjuvant therapy

**Fig. 4:**
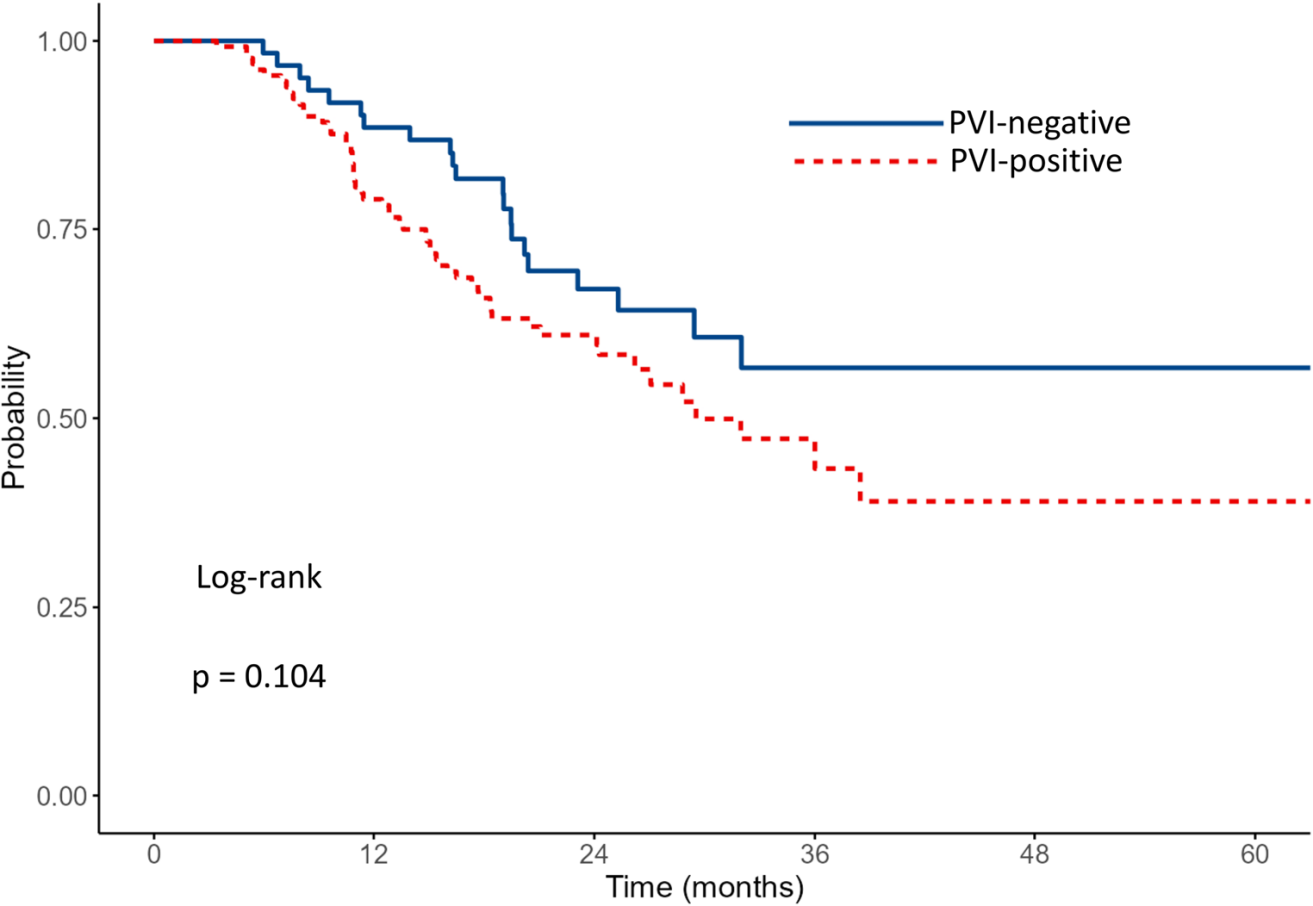
Kaplan–Meier survival curves comparing OS between Adjuvant-treated patients with and without PVI

The median OS for the entire cohort was 25.1 months. Survival outcomes according to PVI status and adjuvant therapy are shown in Table 2. Patients without PVI had a median OS of 32 months (95% CI: 23.7–NA) vs. 21.1 months of those with PVI (95% CI: 16.1–29.5) (*p* = 0.037)​. Data are shown in Fig. 1. The 1- and 3-year survival rates were 79.7% and 47.7% for patients without PVI, and 65.7% and 35.3% for patients with PVI, respectively. Among patients without PVI (*n* = 99), those who received adjuvant chemotherapy (*n* = 69) had a median OS that was not reached (lower bound of 95% CI: 29.4 months), whereas the median OS was 27.5 months (95% CI: 10.1–NA) in those who did not receive adjuvant therapy (*n* = 30) (*p* = 0.015)​. Data are shown in Fig. 2. The 1- and 3-year survival rates were 88.5% and 56.7% in the adjuvant therapy group, compared to 58.3% and 38.3% in the non-adjuvant therapy group. Among patients with PVI (*n* = 196), the median OS was 29.5 months (95% CI: 24.3–NA) with adjuvant therapy (*n* = 135) and 7.0 months (95% CI: 6.1–16.1) without (*n* = 61) (*p* < 0.001)​​. Data are shown in Fig. 3. The 1- and 3-year survival rates were 78.9% and 43.3% in the adjuvant therapy group, compared to 38.9% and 20.9% in the non-adjuvant therapy group. Among patients who received adjuvant chemotherapy (*n* = 204), survival was comparable between patients without PVI (*n* = 69) and those with PVI (*n* = 135). The median OS was not reached (lower bound of 95% CI: 29.4 months) in the no PVI group and was 29.5 months (95% CI: 24.3–NA) in the PVI group (*p* = 0.104)​. Data are shown in Fig. 4. The 1- and 3-year survival rates were 88.5% and 56.7% for patients without PVI, and 78.9% and 43.3% for patients with PVI.

**Table 2:** Survival outcomes according to pathological venous invasion (PVI) status and adjuvant therapy

Group	*n*	Median OS (months) (95% CI)	1-year survival (%)	3-year survival (%)	*P*
All	295	25.1			
PVI-Negative	99	32 (23.7-NA)	79.7	47.7	0.037
PVI-Positive	196	21.1 (16.1–29.5)	65.7	35.3	
Among PVI-negative					
Adjuvant	69	NR (≥ 29.4)	88.5	56.7	0.015
No Adjuvant	30	27.5 (10.1–NA)	58.3	38.3	
Among PVI-positive					
Adjuvant	135	29.5 (24.3–NA)	78.9	43.3	< 0.001
No Adjuvant	61	7.0 (6.1–16.1)	38.9	20.9	
Among Adjuvant-treated					
PVI-Negative	69	NR (≥ 29.4)	88.5	56.7	0.104
PVI-Positive	135	29.5 (24.3–NA)	78.9	43.3	

*OS* indicates Overall Survival, *CI* Confidence Interval, *PVI* Pathologically Confirmed Venous Infiltration, *NA* Not Available (confidence interval upper bound not reached), *NR* Not Reached

### Predictors of overall survival

Univariable and multivariable analyses are displayed in Table 3. The univariable Cox regression analysis identified several predictors OS including age, lymph node status, PVI and administration of adjuvant therapy. The type of surgical procedure was also explored in the univariable analysis. DP was not associated with overall survival compared with PD (HR 1.25, 95% CI 0.75–2.09; *p* = 0.395), whereas TP was associated with worse survival (HR 2.24, 95% CI 1.37–3.68; *p* = 0.001). In the multivariable analysis, independent predictors of OS were age ≥ 75 years (HR 1.93; 95% CI: 1.12–3.32; *p* = 0.017), N2 status (HR 1.89; 95% CI: 1.26–2.83; *p* = 0.002), PVI (HR 1.67; 95% CI: 1.07–2.59; *p* = 0.024), TP (2.42, 95% CI 1.42–4.13; *p* = 0.001) and receipt of adjuvant chemotherapy as a protective one (HR 0.33; 95% CI: 0.22–0.49; *p* < 0.001).

**Table 3:** Univariable and multivariable cox proportional hazards regression on overall survival

Characteristics	Univariable			Multivariable		
	HR	95% CI for HR	*P*	HR	95% CI for HR	*P*
Female Sex	0.93	(0.66–1.29)	0.666			
ASA Score ≥ 3	1.81	(1.27–2.56)	**0.001**	1.35	(0.87–2.11)	0.182
Age at Diagnosis ≥ 75y	2.00	(1.34–2.99)	**0.001**	1.93	(1.12–3.32)	**0.017**
Preop Ca 19.9 > 500 U/mL	1.34	(0.93–1.93)	0.120			
Tumor Diameter ≥ 40 mm	1.38	(0.99–1.91)	0.057			
N Status						
N0	0.86	(0.57–1.30)	0.486			
N1	1 (ref)					
N2	1.76	(1.20–2.58)	**0.004**	1.89	(1.26–2.83)	**0.002**
AJCC Stage						
I	0.67	(0.34–1.30)	0.236			
II	1 (ref)					
III	1.31	(0.93–1.85)	0.117			
R1	0.97	(0.70–1.35)	0.860			
Type of Resection						
Pancreaticoduodenectomy	1 (ref)					
Distal Pancreatectomy	1.25	(0.75–2.09)	0.395			
Total Pancreatectomy	2.24	(1.37–3.68)	**0.001**	2.42	(1.42-4-13)	0.001
PVI	1.49	(1.02–2.17)	**0.037**	1.67	(1.07–2.59)	**0.024**
Adjuvant Therapy	0.33	(0.22–0.47)	**< 0.001**	0.33	(0.22–0.49)	**< 0.001**

*ASA* indicates American Society of Anesthesiologists, *AJCC* American Joint Committee on Cancer, *PVI* Pathologically Confirmed Venous Infiltration

## Discussion

Once a venous resection is deemed necessary in the setting of an upfront surgery for PDAC, the actual involvement of the resected vessel is confirmed at pathology only in 66% of cases. Though, true vascular involvement is a key prognostic factor, as survival is significantly impaired in its presence [17–19]. However, the subsequent administration of adjuvant chemotherapy can ameliorate long-term outcomes as in cases where the venous infiltration is absent [11, 20]. These findings highlight the dual role of PVI as both a biological marker of aggressive disease and a therapeutic turning point. While its presence worsens prognosis, it also defines a subgroup of patients that most benefits from subsequent systemic treatment [20, 25].

Several previous studies have addressed the issue of true vascular involvement in pancreas resections, reporting rates between 55% and 70% in patients undergoing PVR [6, 10, 19]. This variability likely reflects institutional differences in intraoperative judgment, pathological protocols, and definitions of venous invasion [16]. The present series is in line with these data, underscoring how intraoperative suspicion frequently overestimates the actual invasion [6, 19]. Despite advances in high-resolution imaging, preoperative staging often fails to reliably distinguish between inflammatory or fibrotic adhesions and true tumor infiltration [26]. This diagnostic limitation is further complicated by the inherent subjectivity in the interpretation of radiologic findings, as demonstrated by Giannone et al., who reported substantial interobserver variability in assigning resectability status, even among experienced surgeons and radiologists across high-volume centers [27]. Their multicenter prospective study underscores that resectability is still, to a large extent, “in the eye of the observer,” challenging the reproducibility of vascular involvement assessments and, consequently, the reliability of treatment decisions based solely on preoperative imaging. This discrepancy supports the rationale for proceeding with venous resection when technically feasible, as oncologic radicality can still be achieved even in patients who ultimately do not harbour true vascular invasion. Importantly, these findings should not be interpreted as discouraging venous resection when vascular involvement is suspected. In experienced high-volume centers, venous resection represents a safe and widely accepted strategy to achieve margin-negative resection when tumor adherence to the SMV/PV axis is encountered [11, 19].

Moreover, the current series highlights the crucial role played by the administration of adjuvant chemotherapy in this setting, which can provide longer survival when actual vascular infiltration is found [20]. Previous reports have shown that patients with PVI receiving systemic therapy had significantly improved outcomes compared to those who did not, sometimes even achieving survival rates comparable to patients without PVI [19, 25]. These data reinforce a treatment paradigm in which anatomical complexity alone does not dictate prognosis. Instead, tumor biology and treatment completion, particularly the delivery of effective systemic therapy, emerge as dominant drivers of survival [8].

In light of the 34% resected veins not affected by tumor at final pathology (patients without PVI) [19, 28], it is important to underline how the morbidity of vascular resection should be maintained at a low threshold. This represents a crucial factor, as it implies the likely access to adjuvant chemotherapy [29, 30]. Other previous reports have attested that postoperative complications significantly reduce the probability of completing systemic therapy, which in turn negatively affects long-term outcomes [30, 31]. Our series confirmed acceptable morbidity and mortality rates consistent with those reported by high-volume centers, and comparable to standard PD without vascular resection [11, 29]. The setting where such vascular resections are performed is a key factor, namely high-volume centers with expertise in pancreas and vascular surgery. Here surgical morbidity is minimized, and the therapeutic window for adjuvant treatment is likely preserved [26, 29, 32].

This study has several limitations to be highlighted. First, the retrospective nature of the analysis introduces potential biases in patient selection and data completeness. Second, there was inter-institutional variability in surgical strategies, pathological assessment, and chemotherapy regimens [19, 29]. Third, the depth of venous wall invasion (e.g., adventitia, media, or intima) was not systematically reported across the participating institutions and therefore could not be analyzed. Fourth, margin status was recorded according to the standard R classification, but the specific involved margin was not consistently documented across all participating centers. Fifth, we were unable to evaluate disease-free survival, patterns of recurrence, or completion of adjuvant chemotherapy. Lastly, the study exclusively analyzed patients undergoing upfront resection and did not include those treated with neoadjuvant therapy. While growing evidence supports the use of neoadjuvant treatment to improve R0 resection rates and select biologically favourable tumors [7, 25, 33–35], many patients still undergo immediate surgery, either due to resectable status at diagnosis or contraindications to preoperative therapy [20, 36]. In such contexts, our data provide clinically meaningful insights into the prognostic role of PVI and the impact of adjuvant chemotherapy.

## Conclusions

In conclusion, PVI is an independent predictor of poor survival in patients undergoing upfront pancreatectomy with venous resection for PDAC. However, its adverse prognostic impact is substantially attenuated by the administration of adjuvant chemotherapy. The non-infrequent absence of true PVI in resected specimens highlights the limited accuracy of preoperative imaging and intraoperative assessment of even experienced surgeons in identifying true vascular invasion. These findings emphasize the importance of minimizing postoperative morbidity to preserve access to systemic therapy in patients with aggressive tumor biology.

## Electronic Supplementary Material

Below is the link to the electronic supplementary material.

 Supplementary Material 1 (DOCX 20.3 KB)

## Data Availability

The datasets generated and/or analyzed during the current study are available from the corresponding author on reasonable request.
