# Supplementary material for: Prognostic impact of pathologically confirmed venous infiltration during upfront pancreatectomy: multicenter survival analysis
Source: Langenbecks Arch Surg. 2026 Apr 9;411(1):141. doi: 10.1007/s00423-026-04041-2 (PMC13171999; doi:10.1007/s00423-026-04041-2)
Supplement: Supplementary file 1 — Supplementary Material 1 (DOCX 20.3 KB) [file 423_2026_4041_MOESM1_ESM.docx]

| **Supplementary Table** Perioperative Characteristics Stratified by Pathological Confirmed Venous Infiltration (2015-2023) (N=295) | | |  |
| --- | --- | --- | --- |
| **Characteristics** | **PVI-positive patients, n (%) (N=196)** | **PVI-negative pateints, n (%) (N=99)** |  |
|  |  |  |  |
| ***Preoperative*** |  |  |  |
| Age, Median (IQR), y | 65 (14) | 66 (13) |  |
| Female Sex, n (%) | 93 (47) | 49 (49) |  |
| ASA Score ≥3, n (%) | 49 (26) | 37 (40) |  |
| Preoperative biliary drainage, n (%) | 22 (11) | 14 (14) |  |
| Ca 19.9, Median (IQR), U/mL | 180 (560) | 174 (425) |  |
| Tumor Resectability (NCCN), n (%): |  |  |  |
| Resectable | 159 (81) | 80 (81) |  |
| Borderline Resectable | 37 (19) | 19 (19) |  |
| Lesion Site, n (%): |  |  |  |
| Head | 165 (84) | 72 (73) |  |
| Istmus | 2 (1) | 2 (2) |  |
| Body-Tail | 24 (12) | 19 (19) |  |
| Multifocal | 5 (3) | 6 (6) |  |
| ***Intraoperative*** |  |  |  |
| Surgery Type, n (%): |  |  |  |
| Pancreatoduodenectomy | 143 (73) | 63 (64) |  |
| Distal Pancreatectomy | 21 (11) | 18 (18) |  |
| Total Pancreatectomy | 32 (16) | 18 (18) |  |
| Operative Time, Median (IQR), min | 330 (275) | 335 (124) |  |
| Venous Resection, n (%): |  |  |  |
| Tangential | 39 (20) | 33 (33) |  |
| Segmental | 157 (80) | 66 (67) |  |
| ISGPS Type, n (%): |  |  |  |
| I. (Tangential with Primary Closure) | 22 (11) | 26 (26) |  |
| II. (Tangential with Peritoneal Patch) | 17 (9) | 7 (7) |  |
| III. (T-T with Primary Anastomosis) | 139 (71) | 63 (64) |  |
| IV. (T-T with Interposition Graft) | 18 (9) | 3 (3) |  |
| Site of Anastomosis, n (%): |  |  |  |
| PV-PV | 23 (12) | 17 (17) |  |
| SMV-SMV | 59 (30) | 28 (28) |  |
| PV-SMV | 114 (58) | 54 (55) |  |
| Intraoperative Heparin (Reconstruction Phase), n (%) | 176 (90) | 79 (80) |  |
| ***Pathology*** |  |  |  |
| Tumor Diameter, Median (IQR), mm | 35 (30) | 35 (15) |  |
| AJCC Stage, n (%): |  |  |  |
| IA | 2 (1) | 2 (2) |  |
| IB | 15 (8) | 15 (15) |  |
| IIA | 17 (9) | 36 (36) |  |
| IIB | 65 (33) | 35 (36) |  |
| III | 95 (46) | 11 (11) |  |
| N/A | 2 (1) | 0 (0) |  |
| Number of Harvested Lymphnodes, Median (IQR) | 20 (15) | 20 (14) |  |
| Lymphnode Ratio, Median (IQR), % | 10 (20) | 6 (18) |  |
| N Status, n (%): |  |  |  |
| N0 | 51 (26) | 33 (33) |  |
| N1 | 95 (49) | 46 (47) |  |
| N2 | 50 (26) | 20 (20) |  |
| R Status, n (%): |  |  |  |
| R0 | 78 (40) | 45 (45) |  |
| R1 | 118 (60) | 54 (55) |  |
| Vein Invasion Confirmed by Pathology, n (%) | 196 (100) | 0 (0) |  |
| ***Postoperative*** |  |  |  |
| Major Morbidity (Clavien-Dindo ≥3), n (%) | 61 (31) | 37 (37) |  |
| Mortality (30-days/in-hospital), n (%) | 4 (2) | 2 (2) |  |
| Mortality (90-days), n (%) | 13 (7) | 4 (4) |  |
| Adjuvant Therapy, n (%) | 135 (69) | 69 (70) |  |
| Gemcitabine-based regimens | 72 (55) | 44 (64) |  |
| Fluoropyrimidine-based regimens | 23 (17) | 13 (19) |  |
| FOLFIRINOX-based regimens | 6 (5) | 12 (17) |  |
| Other regimens | 34 (23) | 0 (0) |  |
| ASA indicates American Society of Anesthesiologists; ISGPS, International Study Group for Pancreatic Surgery; PV, Portal Vein; SMV, Superior Mesenteric Vein; AJCC, American Joint Committee on Cancer. | | |  |
